# Complexity and Variability of Gut Commensal Microbiota in Polyphagous Lepidopteran Larvae

**DOI:** 10.1371/journal.pone.0036978

**Published:** 2012-07-17

**Authors:** Xiaoshu Tang, Dalial Freitak, Heiko Vogel, Liyan Ping, Yongqi Shao, Erika Arias Cordero, Gary Andersen, Martin Westermann, David G. Heckel, Wilhelm Boland

**Affiliations:** 1 Department of Bioorganic Chemistry, Max Planck Institute for Chemical Ecology, Jena, Germany; 2 Department of Entomology, Max Planck Institute for Chemical Ecology, Jena, Germany; 3 Center for Environmental Biology and Molecular Microbial Ecology, Lawrence Berkeley National Laboratory, Berkeley, California, United States of America; 4 Centre of Electron Microscopy, The University Hospital, Friedrich Schiller University of Jena, Jena, Germany; University of Utah, United States of America

## Abstract

**Background:**

The gut of most insects harbours nonpathogenic microorganisms. Recent work suggests that gut microbiota not only provide nutrients, but also involve in the development and maintenance of the host immune system. However, the complexity, dynamics and types of interactions between the insect hosts and their gut microbiota are far from being well understood.

**Methods/Principal Findings:**

To determine the composition of the gut microbiota of two lepidopteran pests, *Spodoptera littoralis* and *Helicoverpa armigera*, we applied cultivation-independent techniques based on 16S rRNA gene sequencing and microarray. The two insect species were very similar regarding high abundant bacterial families. Different bacteria colonize different niches within the gut. A core community, consisting of Enterococci, Lactobacilli, Clostridia, *etc*. was revealed in the insect larvae. These bacteria are constantly present in the digestion tract at relatively high frequency despite that developmental stage and diet had a great impact on shaping the bacterial communities. Some low-abundant species might become dominant upon loading external disturbances; the core community, however, did not change significantly. Clearly the insect gut selects for particular bacterial phylotypes.

**Conclusions:**

Because of their importance as agricultural pests, phytophagous Lepidopterans are widely used as experimental models in ecological and physiological studies. Our results demonstrated that a core microbial community exists in the insect gut, which may contribute to the host physiology. Host physiology and food, nevertheless, significantly influence some fringe bacterial species in the gut. The gut microbiota might also serve as a reservoir of microorganisms for ever-changing environments. Understanding these interactions might pave the way for developing novel pest control strategies.

## Introduction

Microorganisms play important and often essential roles in the growth and development of insect species. Many insects that harbour endosymbionts depend on them for reproduction, digestion, supply of essential nutrients and pheromone production, etc. [1], [2]. The bacteria in the gut of some specialized niche feeders, such as termites and aphids, have attracted wide attention because of the microbial enzymes achieving particular biochemical transformations [3], [4], [5]. However, relatively little is known about insects feeding on foliage, where no strict symbiotic interaction has been proposed so far. In fact, most lepidopteran larvae are herbivores [6], [7] and their gut content (food bolus) is not sterile [8]. Indigenous gut bacteria of lepidopteran and other insects have been found to detoxify harmful secondary metabolites [9] and to protect the host against the colonization of pathogens [8]. They are also involved in formation of the aggregation pheromones of locusts [10], maintenance of the host fitness [11], [12] and the homeostasis of plant defense elicitors in certain lepidopteran larvae [13], [14], [15].

For a long time, studying insect gut microbiota was mainly performed by cultivation and isolation. These studies formed the basis of our current understanding but often led to a biased description [8]. Less than half of the bacterial phylotypes identified with terminal-restriction fragment-length polymorphism of 16S rRNA genes from gypsy moth (*Lymantria dispar*) were viable on Petri dishes [16]. None of the bacteria isolated from the laboratory-bred tobacco hornworm (*Manduca sexta*) [17] belong to the abundant phylotypes revealed by PCR-single-strand conformation polymorphism of the 16S rRNA genes [18]. A denaturing gradient gel electrophoresis coupled with 16S rRNA gene sequencing has revealed that 72% midgut bacteria of the “old world” cotton bollworm (*Helicoverpa armigera*) shared less than 98% sequence identities to known species [19].

The larvae of African cotton leafworm (*Spodoptera littoralis*) and the cotton bollworm (Lepidoptera; Noctuidae) are generalist herbivores and devastating agricultural pests, feeding on more than a hundred plant species [6]. The uptake food passes through the larval gut quickly, normally within a few hours. Whether autochthonous bacterial strains exist in these insect guts is largely unknown [8]. Here we ask the following questions: i) the taxonomic composition of bacteria living in lepidopteran larval gut; ii) the dynamics of gut microbiota in the course of larval development; iii) the influence of diet on gut microbiota.

## Results

### Bacteria Enumeration

Both *S. littoralis* and *H. armigera* were maintained in the laboratory on heat- and UV-sterilized artificial diet [15]. To rule out the possibility that laboratory conditions have long-term effects on the midgut bacterial community, we compared the *H. armigera* strain TWB that was collected in 2004 in Australia with the strain HELIVI that has been maintained under artificial condition for many years. However, no significant difference between the two *H. armigera* strains was observed.

**Figure 1:**
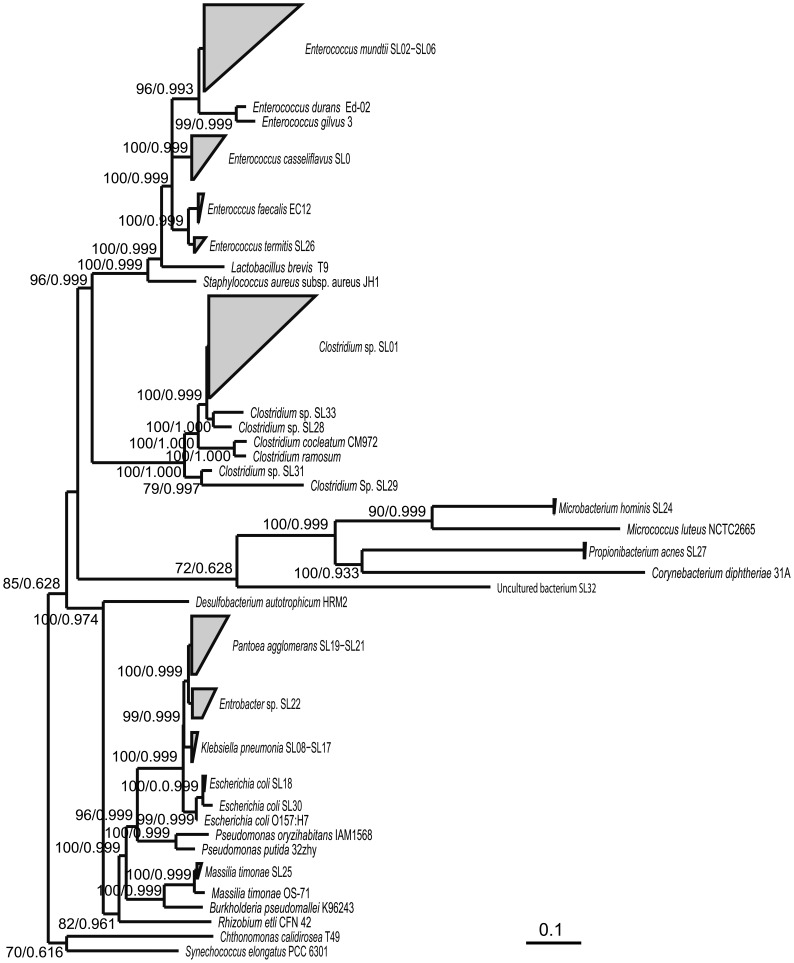
Phylogenetic tree of bacterial divisions retrieved from *S. littoralis* larval gut based on sequence similarity. The 16S rRNA gene sequence of the cyanobacterium *Synechococcus elongatus* PCC 6301 (NC_006576.1) and the Armatimonadetes *Chthonomonas calidirosea* T49 (AM749780.1) were used as the out groups. A detailed description of the phylotypes and accession numbers of the most closely related reference sequences can be found in Table S1. The accession number of the other reference sequences are: *Enterococcus durans* Ed-02 (HM130537.1), *Lactobacillus brevis* T9 (JQ301799.1), *Staphylococcus aureus* subsp. aureus JH1(CP000736.1), *Micrococcus luteus* NCTC2665 (CP001628.1), *Corynebacterium diphtheriae* 31A (CP003206.1), *Burkholderia pseudomallei* K96243 (NC_006350.1), *Rhizobium etli* CFN 42 (CP000133.1), *Desulfobacterium autotrophicum* HRM2 (CP001087.1). The two digit bootstrap number and the three decimal posterior probabilities are shown on major nodes. The bottom bar represents substitution rate per site.

By cloning and sequencing PCR products, we obtained 1473 high-quality bacterial 16S rRNA gene sequences from the *S. littoralis* gut (Figure 1) and 1245 from the *H. armigera* gut. Most of the 18 operational taxonomic units (OTUs) in *S. littoralis* larvae can be classified to known genus based on 99.5% similarity threshold (Table S1). If the sequence is highly similar to one known species, it was named after that species; if the sequence shares equal similarity to two or more species belonging to the same genus, it was regarded as an unknown species of the genus. In addition, sequence heterogeneity exists in several species, which might be attributed to strains or ecotypes. *Clostridium* and *Enterococcus* constitute 42.2% and 42.3% of the final dataset, respectively (Figure 1). Enterobacteriaceae represent the remaining 14.6%. Most of the dominant species in *H. armigera* larvae were identical to those found in *S. littoralis* (Table S2). Furthermore, we could not detect any Archaea in the insect samples.

### Spatial Distribution

**Figure 2:**
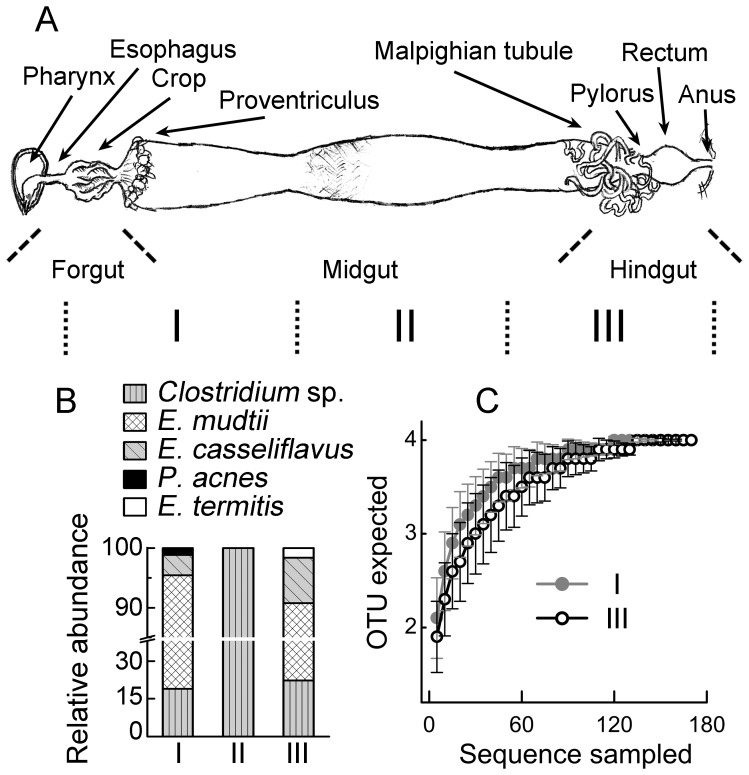
Change of bacterial composition along the digestive tract of 5th-instar larvae of artificial food-feeding *S. littoralis*. (a), The structure of the alimentary canal. The digestive tract was cut into three segments (I, II, and III) for sampling as indicated by the dotted lines. (b), Relative abundance of bacteria in the three segments revealed by cloning and sequencing. (c), Rarefaction curves of the bacterial diversity in gut section I and section III.

**Figure 3:**
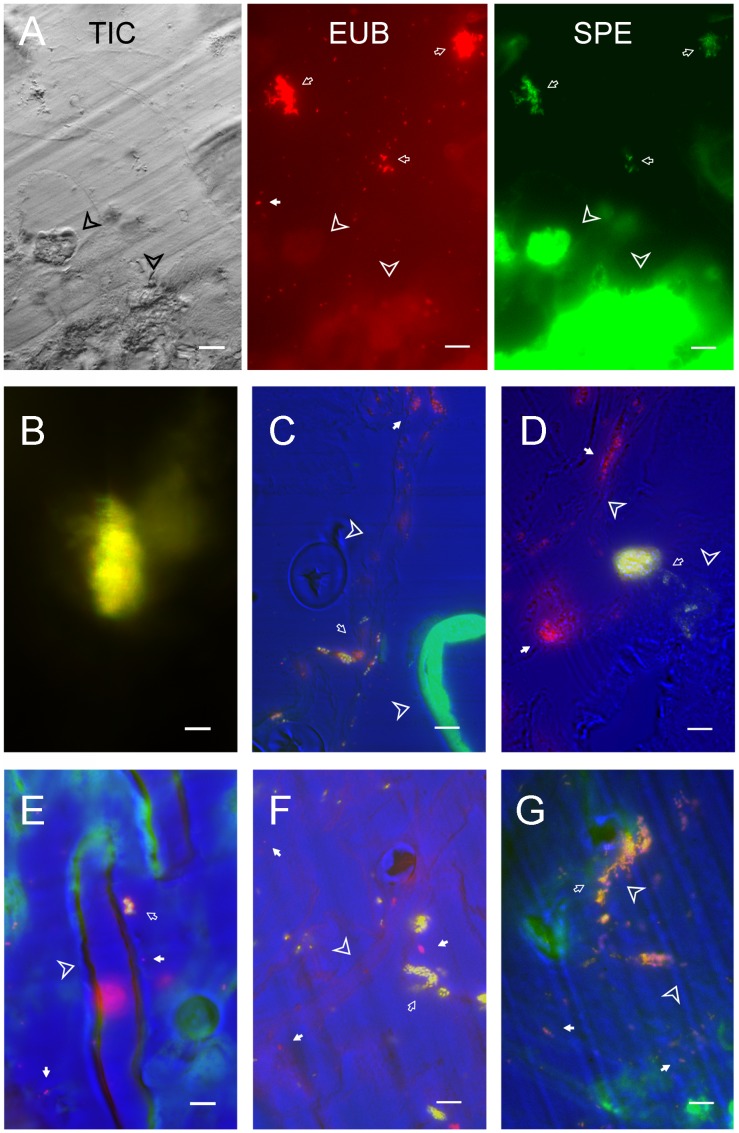
Bacterial localization in the gut of S. littoralis larvae with Fluorescent In Situ Hybridization. Scale bar equals 10 µm. A, Detection of *Clostridium* sp. In the midgut. The three images shown are TIC image, fluorescent image of universal probe (EUB, red) and of specific probe (SPE, green). B to G are merged images of TIC, EUB and SPE. The bacteria detected only with universal probe are red, and the bacterial with both probes are green. B, a large aggregate of *Clostridium* sp. deep in the gut lumen. C, Detection of *E. mundtii*. D, Detection of *E. casseliflavus*. E, *P. acnes* in the midgut. F, *E. coli* detected in the midgut; G, *K. pneumonia* detected in the midgut. Bacteria detected only by universal probe are highlighted with white arrows; Bacteria stained by sequence-specific probes are pointed by open arrows. Insect tissue is indicated by arrow heads.

In Lepidoptera, the larval alimentary canal is composed of three morphologically distinguishable segments [7]: the foregut and the hindgut derived from ectodermal ingrowth and the midgut from the endoderm (Figure 2A). For microbiota analysis, the gut of 5th-instar *S. littoralis* larvae feeding on artificial diet was cut into three segments at the two visible constricting sites on the midgut. In section I, *E. mundtii* is the most dominant species, whereas in section III, *E. casseliflavus* is more dominant. *P. acnes* was only found in section I, and *E. termitis* was only identified in section III. Only one species, namely *Clostridium* sp. SL01 was detected in section II. Rarefaction analyses confirmed that the sequencing is deep enough to reveal high abundance species in section I and III (Figure 2C). Fluorescent in situ hybridization (FISH) using probes designed from the cloned 16S rRNA gene sequences (Table S3) revealed that *Clostridium* sp. SL01 form large aggregates in the deep anoxic area of the food bolus, and small satellite aggregates already exist at 50 µm away from the gut wall. Other species attached to the gut peritrophic membrane (Figure 3).

### Temporal Variation

**Figure 4:**
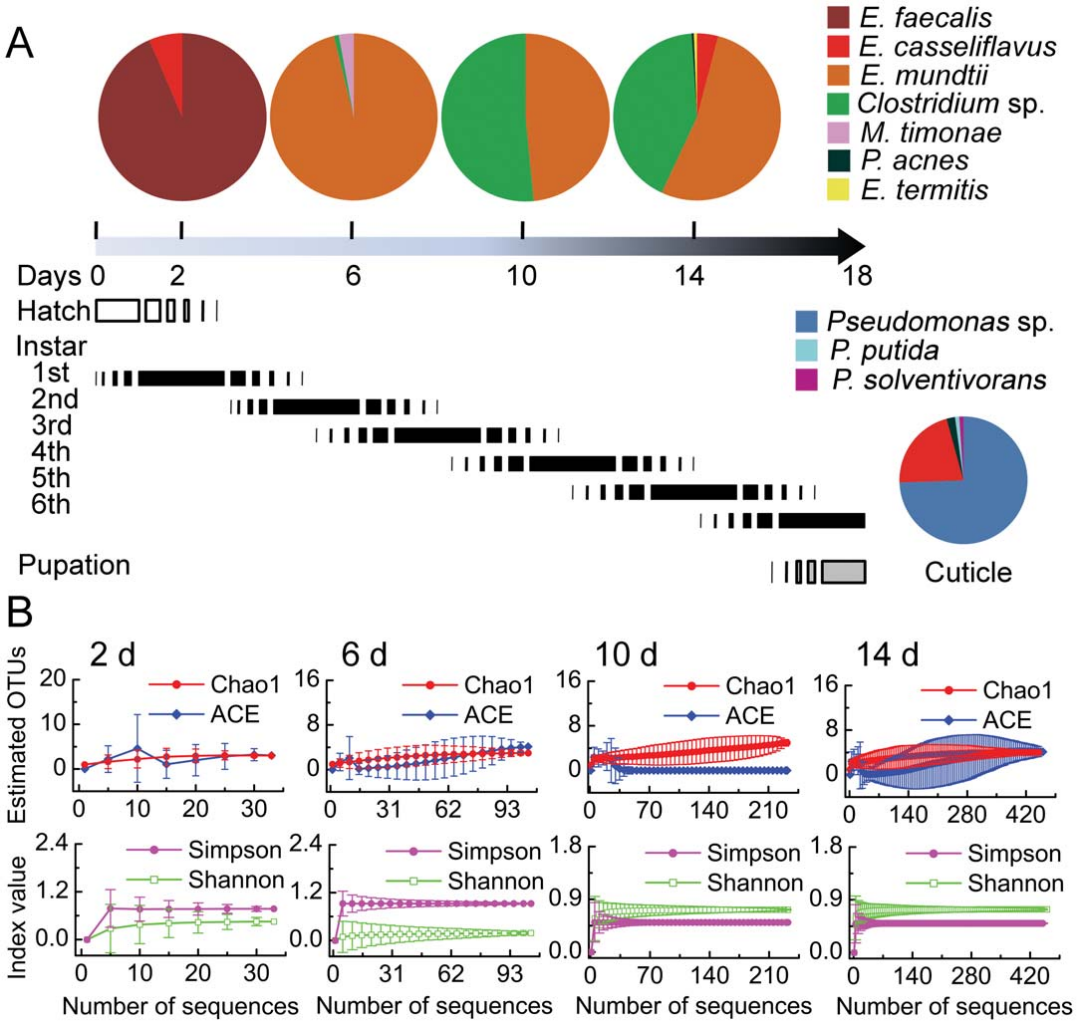
Different gut bacterial community structures in *S. littoralis* larvae of different instars feeding on artificial diet. A, The bacterial community compositions detected by cloning and sequencing from insects that are 2-days (n = 33), 6-days (n = 104), 10-days (n = 232), and 14-days (n = 490). The arrow represents the life span of an *S. littoralis* larva. The developmental stages, hatch, pupation, and larval instars are represented by bars. The inset shows the relative abundance of bacteria detected on the epithelium of 10-day old larvae (n = 94). B, The rarefaction curves of the richness indices Chao1 and ACE, and the diversity indices Shannnon and Simpson based on sequences retrieved from larvae. Indices were calculated using 95% confidence level and 0.03 distance cutoff for OUT clustering.

In the course of larval development, the body length of *S. littoralis* larvae increases from 1.5 mm to ca. 40 mm, and the diameter of its gut increases from 0.5 mm to ca. 7 mm. We monitored the change of dominant species at different instars feeding on artificial diet. The microbiota of the freshly emerged larvae mainly comprised *E. faecalis* and *E. casseliflavus* (Figure 4A). *E. casseliflavus* was also detected on the eggs (data not shown). In older larvae, bacterial diversity increased and *E. mundtii* became very abundant. *E. casseliflavus* was no longer detectable by sequencing but was found with the more sensitive PhyloChip (see discussion below). The *Clostridium* sp. began to appear in 6-day-old larvae. On the larval cuticle, 75% bacterial species were *Pseudomonas*, and *E. casseliflavus* was the only gut inhabitant detected. Statistical analysis with two richness indices Chao1 and ACE (abundance-based coverage estimator) and the α-diversity indices Shannon and Simpson supports the conclusion that the composition of the dominant bacteria in *S. littoralis* larval gut is not complex (Figure 4B).

### The Impact of Food

**Figure 5:**
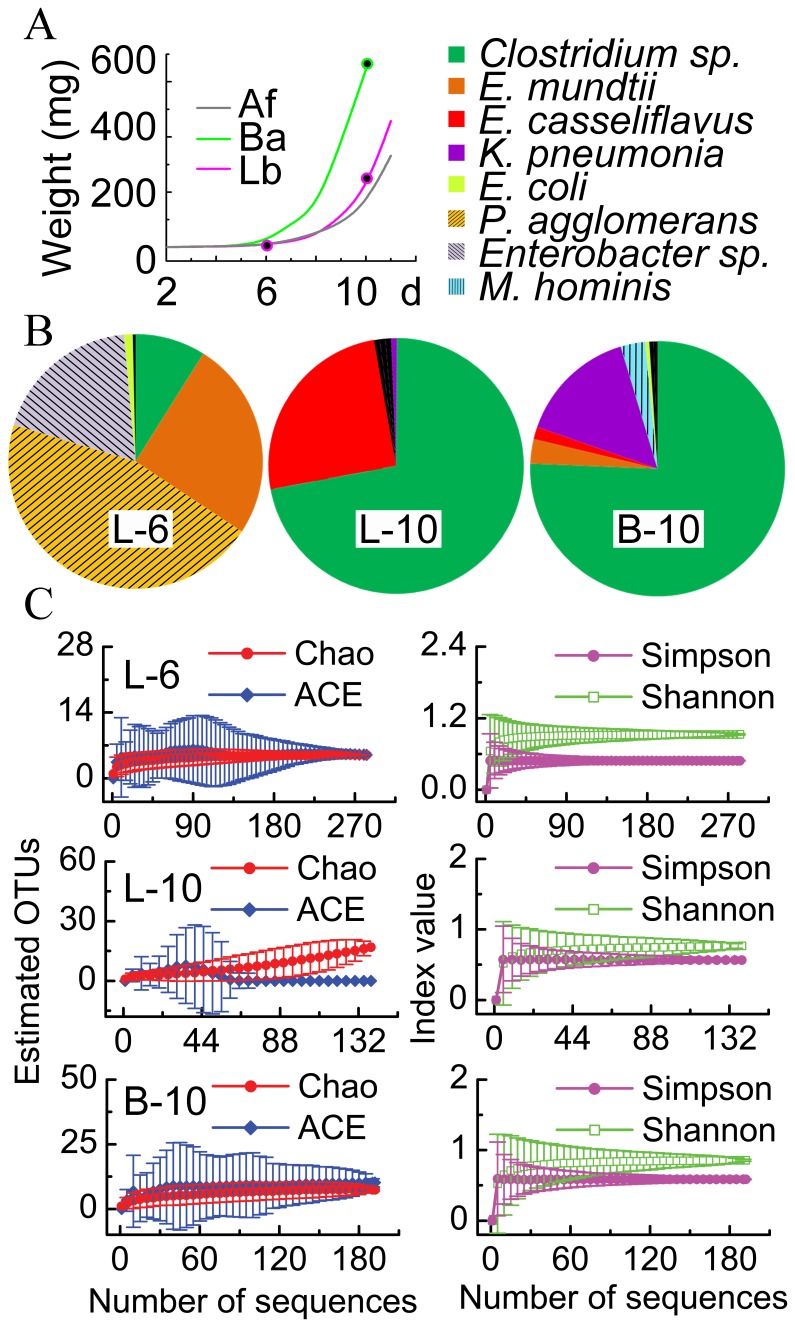
The impact of diet on the gut community in *S. littoralis* larvae revealed by cloning and sequencing. A, Growth curve of the insects. Black dots indicate where the insect gut was sampled. Af, artificial food; Ba, barley; Lb, Lima bean. B, Gut bacterial composition of 6-day-old larvae feeding on Lima bean for 4 days (n = 283, L-6), in 10-day-old larvae feeding on Lima bean (n = 139, L-10), and in the gut of 10-day-old larvae feeding on barley (n = 192, B-10). Case-specific species are shadowed. Singletons are black. C, The rarefaction curves of the richness indices Chao1 and ACE, and the diversity indices Shannnon and Simpson. Indices were calculated using 95% confidence level and 0.03 distance cutoff for OUT clustering.

The influence of food plant on the gut microbiota was also investigated by feeding *S. littoralis* with either Lima bean or barley, and feeding *H. armigera* with cabbage, cotton and tomato. In addition, *E. coli* were doped to the artificial diet of *H. armigera* larvae to mimic food born non-pathogenic bacteria. When the young *S. littoralis* larvae were supplied with the toxic Lima bean containing cyanogenic glycosides [20], a high mortality and a transient growth retardation was observed (Figure 5A). The same phenomenon was observed when *H. armigera* larvae fed on the toxic tomato which contain other alkaloids [21].

The bacterial composition in these plant-feeding insects was dramatically different from artificial diet-feeding insects (compare Figure 4A and Figure 5B). When the larvae suffered from intoxication, their gut microbiota was composed of 25% *E. mundtii* and 50% of *P*. *agglomerans* (Figure 5B). When the larvae recovered after four days, *Clostridia* and *E. casseliflavus* became dominant. In the Barley feeding insects, Clostrida and *K. pneumonia* were most abundant. Even with the slightly more complex microbiota, our sequencing approach is deep enough to cover the dominant species (Figure 5C). A similar pattern was observed when the frass and gut of *H. armigera* larvae were analyzed. Furthermore, in the frass of *H. armigera*, the plant-derived *Burkholderiaceae* sp. was identified in high abundance (Table S2).

### Microarray Analysis

Direct cloning is particularly useful to uncover new and dominant bacterial species, while microarray-based PhyloChip can identify thousands of OTUs simultaneously [22]. The 10-day-old *S. littoralis* larvae that fed on artificial diet, Lima bean, and barley, as well as the *H. armigera* larvae that fed on artificial diet, tomato, and cabbage and the food plants were also subjected to analysis with Affymetrix PhyloChip arrays. 55 OTUs were obtained from *H. armigera* larvae and 46 OTUs from *S. littoralis* larvae. Among them, 39 OTUs belonging to 22 families were common (Table 1). It is worth noting that the microarray OTUs were different from those of the sequencing, because it is based on hierarchical clustering of the fluorescence signals generated with group-specific probes. However, most of the ubiquitous bacterial families were detectable in all larvae and independent of diet. In general, microarray confirmed the results of cloning and sequencing, and some low abundant species were only detected by microarray.

**Table 1:** Bacterial families and genus detected with phylochip in the larvae of H. armiggera (HA) and S. littoralis (SL) and plant.

Phylum/Class	Family/Genus	HA	SL	Plant
Bacteroiddetes	Sphingobacteriaceae	+++	ND	ND
	Flexibacteraceae	+++	+++	ND
	Flavobacteriaceae	ND	ND	ND
	KSA1	+++^1^	+++^1^	+
Acidobacteria	Acidobacteriaceae	ND	+	+
Actinobacteria	Corynebacteriaceae	+	+	ND
	Micrococcaceae	+	+	ND
	Propionibacteriaceae	+	+	ND
	Unclassified	+	+	+
Chloroflexi	Anaerolineae	+++	+++	+
	Thermomicrobia	+	ND	ND
Cyanobacteria	Chloroplasts	+	ND	+++
Deinococcus	Unclassified sf1	+	ND	ND
Firmicutes/Bacilli	Enterococcaceae	+++	+++	ND^2^
	Bacillaceae	+++	+++	ND
	Halobacillaceae	+^3^	+^3^	ND
	Aerococcaceae	+++	ND	ND
	Lactobacillaceae	+++	+++	ND
	Streptococcaceae	+++	+++	ND
Molicutes	Erysipelotrichaceae	+++	+++	ND
Clostridiales	Clostridiaceae	+++	+++	ND
	Lachnospiraceae	+	+	ND
	Catabacter	+++	+++	ND
	Symbiobacteria	ND	ND	+
Planctomycetes	Planctomycetaceae	+^4^	+^4^	ND
	Annamoxales	+++^5^	ND	+++
α-proteobacteria	Caulobacteraceae	+^6^	+^7^	ND
Rhodobacterales	Rhodobacteraceae	+	+	ND
γ-Proteobacteria	Enterobacteriaceae	+^8^	+	ND
	Alteromonadaceae	+^9^	+^9^	ND
δ-Proteobacteria	Desulfovibrionaceae	ND^10^	+	+++
ε-Proteobacteria	Campylobacteraceae	+	ND	ND
Verrucomicrobia	Xiphinematobacteraceae	ND^11^	ND^11^	+
Thermodesulfobacteria	Thermodesulfobacteriaceae	+	ND	ND
OP9/JS1	Unclassified	+++	ND	ND
Unclassified	sf160	+	+	+
	sf156	ND	+	ND
	sf95	ND	+	ND

“+”, low abundance (Z score < 2); “+++”, high abundance (Z score > 2); “ND”, not detected.

1 not found in all insect samples;

2 low abundance only in tomato plant;

3 *S. littoralis* and *H. armigera* possibly contain different species;

4 Found in all plant materials and insects except those feeding on arificial diet;

5 Only detected in plant-feeding *H. armigera*;

6 high abundance in plant feeding larvae and low abundance in artificial diet feeding larvae;

7 only found in one *S. littoralis* sample;

8 not detected in *H. armigera* feeding on cabbage;

9 not in *S. littoralis* eeding on aritficial diet and only in *H. armigera* feeding on artificial diet;

10 high abundance in tomato-feeding *H. armigera*;

11 detected in artificial diet-feeding *S. littoralis* and tomato-feeding *H. armigera*.

## Discussion

The gut microbiota of lepidopteran insects was studied with two complementary and cultivation independent approaches: direct cloning and sequencing that uncovers unknown and dominant bacterial species [23] and a microarray-based approach that monitors low abundant species [22]. Our results clearly showed some dominant bacterial species are shared by two lepidopteran insects. Bacterial species constantly present in the gut are considered as members of the “core set of bacterial community.”

### Core Community

The composition of dominant species of insect gut microbiota can be very simple. A recent survey using 454 sequencing revealed 5dominant OTUs in the gut of the fruit fly (*Drosophila melanogaster*) [24]. In the gut of the gypsy moth and cabbage white butterfly (*Pieris rapae*) were found 23 and 15 OTUs, respectively [16], [25]. We detected 36 dominant OTUs in *S. littoralis* larvae and a similar composition in *H. armigera* larvae. It has been shown that the gut microbiota of laboratory-reared insects is much simpler than those of the insects collected from the field [19], [26].

The fact that insects maintain a stable gut microbiota suggests potential benefits. An *Enterococcus* sp. had been detected in gypsy moth larvae independent of the plant diet [16]. It was the major and the only metabolically active bacterium in the gut and eggs of *Manduca sexta* [18]. *Enterococci* are also prominent in the gut of insects such as *Drosophila*, ground beetle, and desert locust [26], [27], [28]. We detected several *Enterococcus* species in the two lepidopteran larvae, with *E. casseliflavus* being the most widely distributed. The most abundant sequence type in the two lepidopteran larvae belongs to an unknown *Clostridium* species. Clostridia are the dominant bacteria in the guts of termites [5]. We did not detect any Archaea in the lepidopteran insects, in good agreement with the observation on another lepidoteran species *Calyptra thalictri* [29]. Lactobacilli have been detected in the gut of both lepidopteran insects. They were also present in the guts of the fruit fly and the ground beetle [26], [27], [30]. It has been shown that bacteria isolated from other Lepidoptera performed various hydrolytic activity under aerobic conditions [31]. We believe that the core set microbiota would play important roles in host physiology other than digestion.

### Spatial and Temporal Distribution

**Figure 6:**
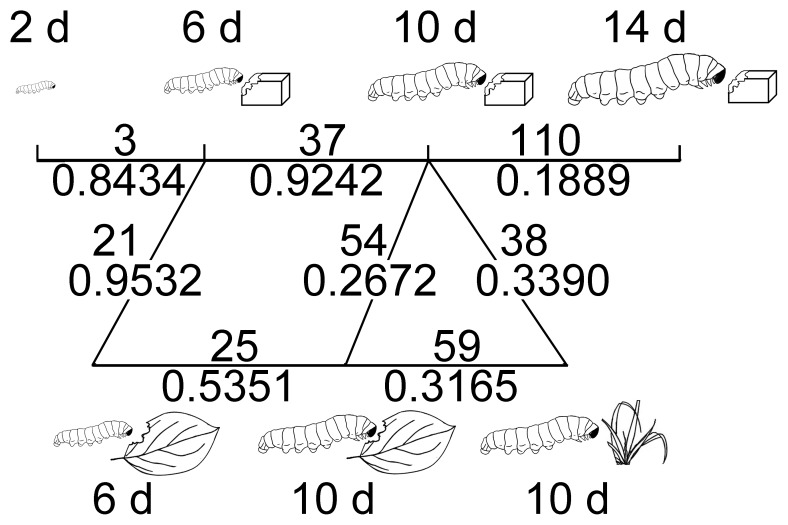
The phylogeny-based β-diversity values between bacterial communities detected in the gut of *S. littoralis* larvae at different instars and after feeding on different diets by cloning and sequencing. The upper values are the parsimony scores and the lower values are the weighted UniFrac scores. Higher score indicates that the two samples are more different on bacterial composition. All significance are lower than 0.001. Artificial food was depicted as cubes; Lima bean as a single leaf; barley as a whole plant.

The tubular lepidopteran midgut is structurally simple, and with a pH gradient from the highly alkaline (ca. 10) anterior end to the nearly neutral posterior ends [14]. The spatial distribution of some bacterial species might reflect their pH tolerance (Figure 2). A strain showing high sequence similarity to *E. termitis* isolated from termite gut was found specifically in the hindgut [32]. *Clostridium* sp. was the most dominant species in the midgut of 6-day-old larva (Figure 4). They were also the most dominant linage in the gut of the European cockchafer, where 100 µm away from the gut all becomes completely anoxic [33]. In the lepidopteran larval gut, *Clostridium* sp. was only detectable about 50 µm inside the gut wall (Figure 3), in accordance with its anaerobic nature. As the insects grew bigger, the ratio of gut volume to the gut surface increased with a factor of D/4 (here D is the diameter of the gut). As a consequence, anaerobic species like *Clostridia* became more dominant. Besides the change of the *Clostridium* sp., the overall composition of the gut microbiota change significantly as the insect ages (Figure 6), suggesting the involvement of other host-derived factor(s) shaping the gut community.

### Impact of Food

Most lepidopteran herbivores are highly polyphagous and naturally exposed to bacteria via food consumption. However, the bacteria on the food plant were very different from those in the guts (Table 1), which are again different from those in frass (Table S2). The alkaline pH, digestion enzymes, reactive oxygen species produced by cells of the gut membrane [34] along with the ionic strength in insect gut generally kill the ingested bacteria [35]. Persisting bacteria might become gut colonizers, or remain as transient passengers [18]. We found examples of all, e.g. *X. campestris* from the artificial diet of *S. littoralis* were not detectable in the insect guts. A bacterium belonging to *Anammoxales* was detected in both plant and insects, while *C. maltaromaticum* was abundant in *H. armigera* frass (Table S2).

The gut bacterial communities in insects feeding on different diet are dramatically deferent (Figure 6). It has been shown that the gut microbial composition was different between crickets feeding on protein-rich diet and those feeding on fiber-rich diet [36]. *P. agglomerans* that was also found in gypsy moth larvae [16] and in locust hindguts [28] was also detectable in our plant-fed larvae (Figure 5). In the *S. littoralis* larvae that ingested Lima bean, many low-abundant species began to bloom. The dominance of some species such as *Enterococci* and *Lactobacilli* can be explained by their cyanide resistance [37]. When a large amount of *E. coli were* ingested, the gut microbiota of *H. armigera* became more complex. Whether this is due to a probiotic effect or dysbacteriosis needs further investigation.

### Conclusions

The comprehensiveness of the current study on microbiota of lepidopteran gut is only comparable by few studies performed on termites [38], and fruit flies [24], [39]. Demonstrating the existence of the core bacterial community established a platform for further evaluation of the tritrophic bacteria-insect-plant interaction. Further research on each individual species as well as genetic and chemical manipulating the insect and bacteria partners will advance our knowledge on the role of lepidopteran gut microbiota far beyond the old assumption as neutral commensals. As microbiota contribute substantially to insect nutritional ecology and other processes, understanding the physiological role of gut microbiota could potentially pave the way for novel pest control strategies.

## Materials and Methods

### Insects and Plants

*S. littoralis* eggs were purchased from Syngenta Crop Protection Münchwilen AG (Münchwilen, Switzerland). The artificial food made of white bean and some essential nutrients was prepared according to [15]. Eggs were hatched at 14°C. Larvae were transferred to room temperature (24°C). Neonatal larvae (400), 2-day-old (400) and 6-day-old (50) larvae were used to prepare the DNA template, while the 10-day-old (20) and 14-day old (7) larvae were dissected, the whole gut was used for DNA preparation. The cuticle of 10-day-old larvae was collected as control. After starvation for 4 hours, larvae were rinsed 3 times alternatively with water and 70% ethanol before dissection. Samples were stored at −20°C before DNA extraction.

*H. armigera* strain TWB (from laboratory stock) and strain HELIAR (Bayer CropScience, Monheim, Germany) were grown on artificial diet or on plants until the beginning of the final instar as described previously [40]. Artificial diet doped with *E. coli* was performed as described before [12]. Midguts (3×5 larvae per diet) were dissected from freeze-killed larvae in ice-cold phosphate-buffered saline solution (PBS), immersed in ice-cold balanced salt solution (BSS) and kept at −20°C.

Tomato (*Solanum lycopersicum*), cabbage (*Brassicae oleraceae*), cotton (*Gossypium hirsutum*), barley (*Hordeum vulgare* subsp. *vulgare* Cultivar: Barke) and lima bean (*Phaseolus lunatus* strain CV_JWBJ A) were cultivated in the greenhouse [20], [37]. Small larvae were reared in a box and supplied with fresh cuttings of plant shoots on a daily basis.

### 16S rRNA Gene Library and Sequencing

Frozen samples were thawed on ice and dried at 45°C in a speedvac (Concentrator 5301, Eppendorf). The dried samples were crushed in a 1.5 ml tube with a plastic pestle. Plant material was ground in liquid nitrogen. DNA was extracted with the PowerSoil™ DNA Isolation Kit (MO BIO Laboratories, Inc., Carlsbad, CA, USA) according to protocol provided by the manufacturer. 240 ng of purified DNA was used as template for a temperature gradient PCR. The primer pairs used to amplify the eubacterial 16S rRNA gene genes were 27f (5′-AGAGTTTGATCCTGGCTCAG-3′) and 1492r (5′-GGTTACCTTGTTACGACTT-3′). The primer pairs used to amplify archaeal sequences were either 4fa (5′-TCCGGTTGATCCTGCCRG-3′) and 1492r or Ar109f (5′-ACKGCTCAGTAACACGT -3′) and Ar912r (5′-CTCCCCCGCCAATTCCTTTA -3′).

The PCR of each sample was performed with 8 tubes. Every tube contained 0.4 mM of each primer, 30 ng template, 300 mM dNTP, 2.5 units Taq polymerase (Invitrogen), and the buffer from the manufacturer. The annealing temperatures on each tube were 47.5°C, 49.0°C, 50.5°C, 52.0°C, 53.5°C, 55.0°C, 56.5°C, and 58.0°C, respectively, to ensure equally efficient amplification of templates with different GC content. Denaturation was achieved by heating at 94°C for 3 min, and followed by 25 cycles: 94°C for 45s, annealing for 30s, and 72°C for 1.5 min. The final elongation was at 72°C for 10 min. Pooled PCR products were concentrated using the QIAquick PCR Purification Kit (QIAGEN GmbH, Hilden, Germany), and further cleaned by running 0.8% agarose gels and cutting out bands of the correct size. Gel slices were purified using the QIAquick Gel Extraction Kit (QIAGEN).

The purified PCR product was cloned with pCR2.1 TOPO TA Cloning Kit (Invitrogen). Colonies were picked and sequenced as described before [41]. DNA sequences were cleaned and assembled with DNASTAR Lasergene software package (DNASTAR, Inc., Madison, WI, USA). Chimeric sequences were discarded. Consensus sequences were used for blast search in databases at the National Center for Biotechnology Information (http://www.ncbi.nlm.nih.gov) and Greengenes (http://greengenes.lbl.gov). Phylogenetic analyses were first performed with ARB 5.3 software package [42]. The obtained tree was compared with the tree generated with the maximum-likelihood algorithm using Phylip3.67 (http://evolution.genetics.washington.edu/phylip.html) and with Bayesian Inference using the software package BEAST v1.6.2 [43]. Rarefaction, the richness indices (abundance-based coverage estimator (ACE), bias-corrected Chao1), the two α-diversity indices (Shannon and Simpson), and the two β-diversity indices (Parsimony and UniFrac) were calculated using the software mothur [44]. The bacterial partial 16S rRNA gene sequences have been deposited at the National Center for Biotechnology Information with accession numbers HQ264061 to HQ264097.

### PhyloChip Analysis

Purified PCR products of 500 ng from each set of pooled samples were used for phylogenetic microarray analysis. Fragmentation and terminal labeling were performed according to the Affymetrix protocol as described in [22]. DNA fragmentation, hybridization and data analysis were performed as previously reported [45]. An OTU was considered to be present in the sample when the positive fraction was larger than 0.90. For each sample, all operational taxonomic units (OTUs) intensity measurements were normalized by a scaling factor such that the overall chip intensity was equal. Raw data output files were analyzed using the Graphical User Interface (LimmaGUI) version of the software Limma and Phylotrac. Each taxon detected was described by a single species.

### Fluorescence in situ Hybridization

5th-instar *S. littoralis* larvae were washed 3 times with 70% ethanol and water. The anesthetized insects were briefly frozen at −20°C and were dissected under microscope. Gut was cut into three pieces (Figure 2A). Different parts of gut were fixed with 4% formaldehyde overnight. After washing 3 times with 1× phosphate buffered saline (PBS), the samples were embedded with Technovit 8100 according to the protocol provided by manufacturer (Heraeus Kulzer GmbH, Wehrheim, Germany). Embedded samples were cut into 5 µm thin sections. The thin sections were mounted on SuperFrost Ultra Plus glass slide (Thermo Scientific) and treated with 5 mg/ml lysozyme for 15 min at 37°C. After washing away the lysozyme, the slide was dried by blowing with air. The side was hybridized with 1.5 µM of each probe (Table S3) in hybridization buffer containing 900 mM NaCl, 0.02 M Tris-HCl (pH8.0), 20% formamide, 1% SDS. Hybridization was performed at 46°C for 4 hours on the Advalytix slide booster (Beckman Coulter Biomedical GmbH, Munich, Germany). Afterward, the slide was washed in 50 ml washing buffer containing 0.02 M Tris-HCl (pH 8.0), 0.2 M NaCl, 0.05 M EDTA, 1% SDS at 48°C for 20 min. Slide was then washed with running water for 30 sec and dried with blowing air. Images were taken with an Axio Imager Z1 microscope (Carl Zeiss) equipped with an AxioCam MRM camera.

## Supporting Information

Table S1 **Bacterial partial 16S rRNA gene sequences cloned from** ***S. littoralis*** **larvae and the BLAST results.**
(DOC)

Table S2 **Bacteria detected in** ***H. armigera*** **larval gut and frass based on cloning and sequencing.**
(DOC)

Table S3 **FISH probes used to detect bacteria in** ***S. littoralis*** **gut.**
(DOC)
