# Supplementary material for: Complexity and Variability of Gut Commensal Microbiota in Polyphagous Lepidopteran Larvae
Source: PLoS One. 2012 Jul 17;7(7):e36978. doi: 10.1371/journal.pone.0036978 (PMC3398904; doi:10.1371/journal.pone.0036978)
Supplement: Table S1 — Bacterial partial 16S rRNA gene sequences cloned from S. littoralis larvae and the BLAST results. (DOC) [file pone.0036978.s001.doc]

**Table S1**. Bacterial partial 16S rDNA gene sequences cloned from *S. littoralis* larvae and the BLAST results.

| Isolation No. | known species | | | Most similar sequences^b^ | | |
| --- | --- | --- | --- | --- | --- | --- |
|  | Organism | Accession No. | % identity | Organism | Accession No. | % identity |
| SL01 | *Clostridium ramosum* | M23731.1 | 92.30 | Soil clone from tallgrass prarie FFCH17703 | EU134663.1 | 99.11 |
| SL02 - SL06^a^ | *Enterococcus mundtii* str. ATCC43186 | AF061013.1 | 99.93 |  |  |  |
| SL07 | *Enterococcus casseliflavus* str. F32 | AF039903.1 | 99.87 |  |  |  |
| SL08 - SL17 ^a^ | *Klebsiella pneumonia* str. 342 | CP000964.1 | 99.93 |  |  |  |
| SL18 | *Escherichia coli* O157:H7 str. EC4115 | NC_011353.1 | 99.87 |  |  |  |
| SL19 - SL21 ^a^ | *Pantoea agglomerans* str. ChDC YP1 | AY691543.1 | 99.73 |  |  |  |
| SL22 | *Enterobacter asburiae* str. J2S4 | EU221358.1 | 99.60 | *Enterobacter* sp. str. J11 | EU099377.1 | 99.80 |
|  | *Pantoea agglomerans* str. ChDC YP1 | AY691543.1 | 99.60 | microbiota antlions | DQ068844.1 | 99.73 |
| SL23 | *Enterococcus faecalis* str. EC-12 | AB154827.1 | 100 |  |  |  |
| SL24 | *Microbacterium hominis* str. DSM 12509 | AM181504.1 | 99.86 | Contaminated soil bacterium rJ6 | AB021324.1 | 100 |
| SL25 | *Massilia timonae* OS-71 | AM237371.1 | 98.78 | Indoor dust clone BF0002D02 | AM697512.1 | 99.12 |
|  |  |  |  | Cave-wall biofilm clone LKC_Acid_11 | EU038009.1 | 99.13 |
| SL26 | *Enterococcus termitis* str. LMG 8895 | AM039968.1 | 99.20 |  |  |  |
| SL27 | *Propionibacterium acnes* #4584 | AB042287.1 | 99.66 | soil microbial clone 227 | DQ158099.1 | 99.66 |
| SL28 | *Clostridium ramosum* | M23731.1 | 90.22 | Soil clone from tallgrass prarie FFCH17703 | EU134663.1 | 97.07 |
| SL29 | *Clostridium piliforme* str. RJ | D14638.1 | 88.43 | Solid waste clone G35_D8_H_B_C11 | EF559167.1 | 89.12 |
| SL30 | *Escherichia coli* K12 str. K-12 | NC_000913.2 | 98.74 |  |  |  |
| SL31 | *Clostridium cocleatum* CM972 | AF028350.1 | 89.67 | Soil clone from tallgrass prarie FFCH17703 | EU134663.1 | 96.20 |
| SL32 | *Dehalococcoides* sp. str. CBDB1 | AF230641.1 | 78.07 | Contaminated soil bacterium TF7 | DQ248299.1 | 98.61 |
| SL33 | *Clostridium ramosum* | M23731.1 | 87.29 | Soil clone from tallgrass prarie FFCH17703 | EU134663.1 | 93.75 |
| SL34 | *Pseudomonas oryzihabitans* str. IAM 1568 | AM262973.1 | 99.86 | *Pseudomonas* sp. CYN01B | AB175661.1 | 99.93 |
|  | *Pseudomonas psychrotolerans* str. C36 | AJ575816.1 | 99.86 |  |  |  |
| SL35 | *Pseudomonas putida* str. 32zhy | AM411059.1 | 99.93 |  |  |  |
| SL36 | *Paracoccus solventivorans* | Y07705.1 | 100 |  |  |  |
| SL37 | *Xanthomonas campestris* pv. *vesicatoria* | AM039952.1 | 100 |  |  |  |

^a^ the consensus sequences of these sequences were used in the blast search. ^b^ only applied to the clones which were not identifiable by comparing to the sequences of known species.
