# Supplementary figures and images for: Complexity and Variability of Gut Commensal Microbiota in Polyphagous Lepidopteran Larvae
Source: PLoS One. 2012 Jul 17;7(7):e36978. doi: 10.1371/journal.pone.0036978 (PMC3398904; doi:10.1371/journal.pone.0036978)

### Table S2. Bacteria detected in *H. armigera* larval gut and frass based on cloning and sequencing.


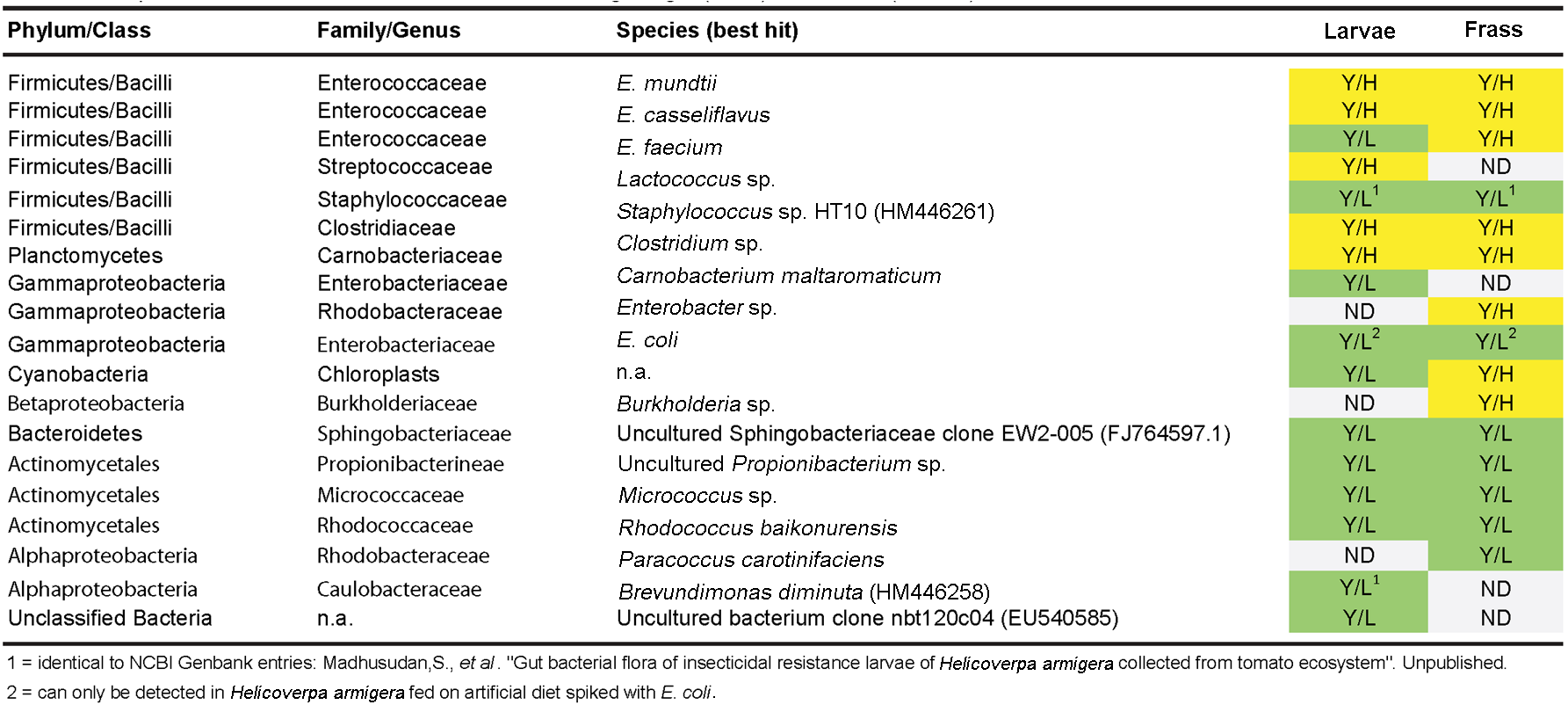

Supplement: Table S2 — Bacteria detected in H. armigera larval gut and frass based on cloning and sequencing. (DOC) [file pone.0036978.s002.doc]
