# Supplementary material for: Complexity and Variability of Gut Commensal Microbiota in Polyphagous Lepidopteran Larvae
Source: PLoS One. 2012 Jul 17;7(7):e36978. doi: 10.1371/journal.pone.0036978 (PMC3398904; doi:10.1371/journal.pone.0036978)
Supplement: Table S3 — FISH probes used to detect bacteria in S. littoralis gut. (DOC) [file pone.0036978.s003.doc]

### Table S3. FISH probes used to detect bacteria in *S. littoralis* gut.

| Probe | Target | Sequence | Labeling |
| --- | --- | --- | --- |
| EUB338 | all | 5’-GCTGCCTCCCGTAGGAGT-3’ | Cy3 |
| sl001 | *Clostridium sp.* | 5'-CACTGATATACCATTTCCTGC-3' | Fluorescein |
| sl002 | *Enterococcus mundtii* | 5'-AGGGGTGAACAGTTACTCTC-3' | Fluorescein |
| sl003 | *Enterococcus casseliflavus* | 5'-GGGATGAACATTTTACTCTCA-3' | Fluorescein |
| sl004 | *Escherichia coli* | 5'-CAATGAGCAAAGGTATTAACTT-3' | Fluorescein |
| sl005 | *Klebsiella pneumonia* | 5'-CAATCGGTGAGGTTATTAAC-3' | Fluorescein |
| sl006 | *Propionibacterium acnes* | 5'-ACTCACGCTTCGTCACAG-3' | Fluorescein |
| Sl007 | *Pantoea agglomerans* | 5'-TGCTGCGGTTATTAACCG-3' | Fluorescein |
